# Acceptability of alcohol-based hand rub during neonatal care in rural Ugandan households: a qualitative study nested within the BabyGel trial

**DOI:** 10.1080/16549716.2026.2727791

**Published:** 2026-09-04

**Authors:** Heidi H. Thorsdalen, Ingunn Marie S. Engebretsen, David Mukunya, Vivian Mutaki, Agnes Napyo, Thorkild Tylleskär, Andrew Weeks, Melf-Jakob Kühl

**Affiliations:** a Centre for International Health, Department of Global Public Health and Primary Care (IGS), Faculty of Medicine, University of Bergen, Bergen, Norway; b Faculty of Health Sciences, Busitema University, Mbale, Uganda; c Department of Nursing, Kabale University, Kabale, Uganda; d Department of Women’s and Children’s Health, University of Liverpool, Liverpool, UK

**Keywords:** Acceptability, Uganda, newborn health, infection prevention and control, hand hygiene

## Abstract

**Background:**

Poor access to water, sanitation and hygiene exacerbates the spread of infections among newborns. The BabyGel cluster randomised trial assessed the effectiveness of a community-level alcohol-based hand rub with a training component in reducing infection or death rates among newborns in Uganda.

**Objective:**

Nested in the BabyGel trial, this study investigated the acceptability of the BabyGel intervention among mothers and household members in Eastern Uganda, using the Theoretical Framework of Acceptability.

**Methods:**

In 2022, we conducted individual semistructured interviews with mothers, and group interviews with mothers and other household members, all recruited from intervention-arm clusters. Thematic analysis combined inductive and deductive coding, structured by the framework’s seven constructs: affective attitude, burden, ethicality, intervention coherence, opportunity costs, perceived effectiveness and self-efficacy.

**Results:**

Twenty mothers and 14 household members participated in 10 individual and 10 small group interviews. Participants found the intervention generally acceptable, appreciating its convenience and perceived protection against infection. Many described hand hygiene changes that persisted beyond the intervention period. However, concerns related to its harmful effects, spiritual beliefs and social tensions with visitors occasionally influenced its use.

**Conclusion:**

Alcohol-based hand rub combined with a training component was generally well accepted, driven by perceived health and practical benefits, although concerns related to safety, belief systems and social dynamics remained. Because data collection coincided with the COVID-19 pandemic, a period of heightened hygiene awareness, the acceptability observed could differ in nonpandemic periods. Culturally adapted education, community engagement and reinforcement of correct use may enhance acceptability and sustainability in similar settings.

## Paper Context

- **Main findings:** Mothers and household members in Eastern Uganda found the community-level alcohol-based hand rub intervention generally acceptable, particularly because of its perceived effectiveness, ease of use and convenience compared with water and soap.
- **Added knowledge:** This study provides in-depth qualitative insights into the opportunities and challenges shaping the acceptability of a household hand hygiene intervention for newborn care, highlighting how cultural beliefs, social norms and perceived risks influence responses to alcohol-based products.
- **Global health impact for policy and action:** The findings indicate generally satisfactory acceptability of alcohol-based hand hygiene interventions in newborn infection prevention strategies in low-resource settings with limited access to water, while underscoring the importance of culturally tailored education and community engagement.

## Background

Poor access to water, sanitation, and hygiene (WASH) increases exposure to infectious pathogens among neonates and children under 5 years of age. Infections such as pneumonia, diarrhoea, sepsis and meningitis remain major causes of mortality, alongside prematurity, birth complications and congenital anomalies [1,2]. Although deaths among children younger than 5 years have slowly declined globally in recent years, reaching 4.8 million in 2023, progress in reducing neonatal mortality has stalled and neonatal deaths now account for almost half of all deaths [1]. In 2023, 2.3 million newborns died within their first month of life [1], with sub-Saharan Africa having the highest neonatal mortality rate of 27 deaths per 1000 live births [3]. Newborns are particularly vulnerable because of their immature immune systems and their dependence on caregivers for essential daily care, increasing the risk of pathogen transmission through household contacts [4].

A recent scoping review found handwashing with soap to be the consistently recommended hand hygiene method in community settings, with alcohol-based hand rub (ABHR) recommended as complement or alternative [5]. A systematic review also found that promoting handwashing with soap reduced diarrhoea risk in young children in low- and middle-income countries by 30% [6], but it remains unclear whether the same benefits are seen in neonates, who have a high burden of infection.

Access to water is necessary for handwashing with soap. However, according to the Demographic and Health Survey in 2022 in Uganda, there is restricted access for many: only 47% of Ugandan rural households had a place for handwashing [7]. Among households with an observed handwashing station, only 36% had both water and soap [7].

These constraints may limit the feasibility of handwashing with soap and water, particularly during newborn care when frequent hand hygiene is required. ABHR has been proposed as an alternative or complement to handwashing in settings where access to water and soap is limited [8–11]. The effectiveness of ABHR is also less dependent on the correct technique than that of handwashing with soap and water [9,12]. However, the potential benefits of ABHR probably depend on its acceptability among intended users [8]. Studies from Sierra Leone and Liberia reported high acceptability of ABHR among health-care workers [10,13]. Similarly, ABHR was deemed acceptable in rural Bangladeshi households, which, like the BabyGel trial, incorporated ABHR distribution, training and instructional posters [11]. While ABHR was valued for its convenience, soap and water remained the preferred option for removing visible dirt [11,13]. In a crossover study of three alcohol-based hand rub formulations, all three were well received [14].

The BabyGel trial evaluated the effectiveness of household ABHR in preventing diarrhoea, sepsis and pneumonia in Ugandan infants up to 3 months [15], with the intervention consisting of three components: (a) training in the last trimester of pregnancy on the use of ABHR; (b) an educational poster (supplementary material) showing recommended use of ABHR before and after birth; and (c) the provision of 6 l of the ABHR product in different containers. Household members were instructed to rub their hands with ABHR until dry before touching the child; women in the study were additionally instructed to use ABHR after toilet use or diaper changes and begin use before birth [15].

Acceptability, the central concept in this study, has been defined as the extent to which people delivering or receiving an intervention consider it appropriate, based on their anticipated or experienced cognitive and emotional responses to it [16]. To assess acceptability systematically, we applied the Theoretical Framework of Acceptability, a multidimensional model comprising seven constructs: affective attitude (how a person feels about the intervention), burden (the effort it requires), ethicality (its fit with personal values), intervention coherence (how well it is understood), opportunity costs (what must be given up to take part), perceived effectiveness (whether it is seen to work) and self-efficacy (confidence in performing the required actions) [16]. Understanding factors influencing acceptability of this intervention may allow for a more contextualised interpretation of trial findings and feasibility in rural settings.

Hygiene promotion initiatives may be shaped by the cultural context and perceived as demeaning if insufficiently adapted. Although ABHR has shown acceptability in several low-resource settings, evidence from rural Uganda remains limited. A BabyGel pilot study [17] found a hand hygiene poster to be well received and associated with high reported adherence [18]. The present study examined mothers’ and household members’ acceptability of the BabyGel intervention as implemented in Eastern Uganda, providing context for interpreting the BabyGel trial outcomes and informing future WASH interventions in similar settings.

## Methods

### Site

This study was embedded in the BabyGel trial. The trial protocol has been published [15], and findings have recently been submitted for publication. The trial was conducted in Nakaloke, Busiu and Budaka in Eastern Uganda and included 5932 pregnant women from 72 clusters (villages) randomised 1:1 to intervention or control. Pregnant women in intervention clusters received the BabyGel intervention, while pregnant women in both arms received a Maama birthing kit and standard prenatal education [19].

### Design and positionality

This qualitative study used a phenomenological approach to explore participants’ experiences, perceptions and meanings related to ABHR use in everyday newborn care. Data were collected through semi-structured in-depth and group interviews with participants allocated to the intervention arm. The design enabled exploration of ABHR acceptability at both household and individual levels [20].

The first author (HHT) is a Norwegian research-track MD candidate without prior qualitative research experience or experience in similar contexts. This outsider position may have influenced her interpretation of the data, particularly in relation to cultural meanings and understandings. To mitigate this, transcripts, codes and themes were discussed with co-authors familiar with the study context. Co-authors include male and female clinicians and researchers (MD, PhD, MD/PhD) with expertise in paediatrics, public health, hand hygiene and qualitative methods. Data collection was conducted by trained male and female research assistants with university-level health science education and prior qualitative experience; one (VM) is a co-author. IMSE, MK, DM and VM reviewed the data and contributed to the analysis. Reporting followed the Consolidated Criteria for Reporting Qualitative Research checklist [21] (Supplement).

### Participants

Women from intervention villages became eligible once they had exited the BabyGel trial, when their infants reached 3 months of age. As participants referred to themselves as ‘mothers’, this term is used throughout. Eligible participants were mothers, aged ≥18 years or emancipated minors, fluent in Lumasaba, Lugwere, Luganda or English, and their adult household members (relatives or close persons).

Participants were purposively selected with assistance from village health teams and approached in person. Sampling aimed to capture variation in participant experiences by including mothers who had completed the intervention at different time points following trial completion, allowing exploration of a range of perspectives on ABHR use. Research assistants subsequently provided study information, invited participation and arranged interviews. All 34 eligible individuals approached for participation agreed to take part in the study. Mirroring the trial’s procedure, all participants, both adults and emancipated minors, received written and oral study information in their language and provided written informed consent [15].

### Data collection

Interviews (individual, pair and group) were conducted between May and December 2022. The interview guide (Supplementary material) covered three topics: ABHR, ABHR training and the BabyGel poster instructing correct ABHR use. To promote consistency across interviewers, research assistants received training on the study objectives prior to data collection. The interview guide was pre-tested and refined through regular team discussions during data collection, allowing emerging topics to be explored. Interviews were conducted in local languages by research assistants with no prior relationship to participants. Some home interviews occurred in the presence of children or other household members.

Interviews were audio-recorded, translated, transcribed into English and de-identified. Field notes were taken during and after interviews. For quality assurance, six randomly selected recordings were independently translated and transcribed by an external transcriber, and the transcripts were subsequently compared for consistency. Individual interviews with mothers were coded ‘M’; pair and group interviews were coded ‘H’ (household number) and ‘HM’ (household member).

Data collection followed an iterative process, with concurrent preliminary analysis until no new themes emerged, consistent with the principle of data saturation. No repeat interviews were conducted. Transcripts were not returned to participants for comment or correction.

### Data analysis

Data were organised in NVivo (version 12) and analysed using the six phases of Braun and Clarke’s reflexive thematic analysis [22–24]: (1) familiarisation through repeated reading of the transcripts; (2) systematic coding, which began inductively by coding participants’ experiences, perceptions and practices close to the data, and then drew deductively on the Theoretical Framework of Acceptability (TFA) and its seven constructs [16]; (3) collating codes into candidate themes; (4) reviewing these against the coded extracts and the full dataset; (5) refining, defining and naming themes; and (6) writing up, selecting illustrative extracts and relating findings to the research question and literature.

The TFA was used as an organising framework rather than as a set of categories into which data were sorted. Themes were generated analytically from the coded data and were subsequently located within the construct they best illuminated. Although HHT led the coding and analysis, the developing codes, themes and interpretations were discussed regularly with co-authors to support reflexivity and keep the findings grounded in the data. Participants did not provide feedback on the findings.

## Results

### Participant characteristics

All 34 interview participants were women with a median age of 27 years (range 17–64 years) (Table 1). The participants included 20 mothers and 14 household members. Ten mothers participated in individual interviews, and 10 mothers, together with household members, participated in group interviews. No eligible participants declined to participate or withdrew after enrolment. The interviews lasted approximately 60 min on average (range 40–80 min).

**Table 1.** Sociodemographic characteristics of the study participants (*N* = 34).

Characteristics	Mothers (n = 20)	%	Household members (n = 14)	%	Total (N = 34)	%
**Age (years)**						
17–24	8	(40)	4	(29)	12	(35)
25–39	12	(60)	5	(36)	17	(50)
40–64	0	(0)	4	(29)	4	(12)
Missing	0	(0)	1	(7)	1	(3)
**Education (years)**						
0–7	14	(70)	8	(57)	22	(65)
8–13	4	(20)	5	(36)	9	(27)
Above 13	2	(10)	0	(0)	2	(6)
Missing	0	(0)	1	(7)	1	(3)
**Religion**						
Muslim	11	(55)	7	(50)	18	(53)
Christian	9	(45)	7	(50)	16	(47)

### Main findings

Reflexive thematic analysis generated 11 themes, presented within the seven constructs of the TFA that framed the analysis (Table 2).

**Table 2.** Themes reflecting mothers’ and household members’ perceptions of the BabyGel intervention by TFA categories.

Construct	Definition	Theme
Affective attitude	How an individual feels about an intervention	Valuing ABHR for comfort, cleanliness and status Fear and mistrust of ABHR as potentially harmful
Burden	The perceived amount of effort that is required to participate in an intervention	ABHR as a convenient alternative to handwashing Challenges and physical discomfort in using ABHR
Ethicality	The extent to which the intervention has good fit with an individual’s value system	Negotiating ABHR against religious, spiritual and cultural beliefs
Intervention coherence	The extent to which the participant understands the intervention and how it works	Hands as a route of infection to a fragile newborn
Opportunity costs	The extent to which benefits, profits or values must be given up to engage in the intervention	Time costs and everyday inconveniences with ABHR use Social costs and strained relationships with visitors
Perceived effectiveness	The extent to which the intervention is perceived as likely to achieve its purpose	Belief that ABHR protects the health of baby and family Doubt about the need for and effectiveness of ABHR
Self-efficacy	The participant’s confidence that they can perform the behaviour(s) required to participate in the intervention	Building new hygiene routines that were not always sustained

### Affective attitude—positive and negative feelings about using ABHR

#### Valuing ABHR for comfort, cleanliness and status

The participants were generally appreciative of ABHR. Using the ABHR created a feeling of comfort. They felt a relaxing sensation after using it and enjoyed feeling clean and protected, believing that the ABHR had removed germs from their hands. They also appreciated how soft their hands felt after using ABHR, which contributed to more frequent use:

I felt good and felt like using it all the time because the sanitizer [ABHR] made the hands soft and fresh. (HM01, H10)

Views on the smell differed. While some participants appreciated it, others found it unpleasant. However, most considered it acceptable, noting that the alcohol scent evaporated quickly. Scepticism related to the smell was more common among visitors and other community members, some of whom were reluctant to use ABHR.

A few mothers also experienced nausea during pregnancy, which they attributed to the smell of ABHR, and some temporarily stopped using it until after giving birth:

I would feel nauseated, and it made me feel like vomiting most times until I got used to it [the smell]. (M05)

Mothers and household members described how possessing ABHR elevated their social status to ‘another level’ (HM01, H07). They considered it expensive and precious, unaffordable for many. Using ABHR signalled that they were able to take care of both themselves and their families and had access to a valued means of disease prevention. Many reported that others looked up to or envied them because they had access to ABHR.

I used to move with it everywhere I would go … everyone would look at me in admiration. (M01)

However, this perceived prestige was not universally viewed positively. Some participants felt the need to explain or defend their use of ABHR to others in the community. Using ABHR in public could be perceived as showing off or implying that others were unclean, occasionally creating social tensions and affecting relationships:

I used sanitizer [ABHR] after shaking her [friend’s hand] and she said ’why would you use sanitizer [ABHR] after shaking my hands … you are looking at me as a dirty one’? (HM01, H09)

There was a clear enthusiasm for ABHR, particularly because it was a novel addition to their hygiene routine. Participants expressed curiosity and excitement about ABHR, as many had never used it but had only heard about it, a factor that appeared to further increase its popularity:

Some pretended to be coming to carry the baby, but in the actual sense, all they wanted was to use some of the sanitizer [ABHR] because they loved it. (M08)

However, for some participants this distribution coincided with the onset of the COVID-19 pandemic, which was accompanied by a large national information and hygiene-awareness campaign. Participants considered COVID-19 to be dangerous and due to their fear of it and of other diseases, many community members wanted to have and use ABHR. Caregivers saw the ABHR as a means to protect both the baby and themselves:

They would quickly use it without any hesitation because they were scared of COVID-19. (M04)

#### Fear and mistrust of ABHR as potentially harmful

While participants predominantly expressed positive feelings towards ABHR, some described fears related to its use and storage. During training, participants were informed that ABHR was flammable, should be kept away from children, not be ingested, not applied to wounds and not used on the baby’s skin. Some therefore considered ABHR poisonous on skin contact, believing it could penetrate the skin and harm internal organs. Others avoided ABHR before eating or cooking because they feared residues could be ingested with food. The baby was perceived as particularly delicate and fragile, and some feared that ABHR could be absorbed and harm the baby before or after birth:

A pregnant woman is not supposed to apply it on the stomach, because if she does, it will penetrate into the womb and affect the baby and other inner organs. (M08)

Managing fears and myths around ABHR, while seeking to benefit from its advantages, a few participants developed their own safety procedures, for example by washing off ABHR remains with water before cooking, eating or breastfeeding.

I knew that it was helpful but to prevent the chemical from getting into the body, I had to wash my hands after using. (M07)

#### Burden—perceived effort required to use ABHR regularly

##### ABHR as a convenient alternative to handwashing

Comparing ABHR to using water and soap, mothers and household members generally considered ABHR more convenient. ABHR was generally easily accessible, with one bottle of ABHR kept in the household and another used for travelling outside the home. Compared to water and soap, ABHR was described as time-saving because participants did not need to fetch water, and it dried quickly on the hands. Some participants were already familiar with hand rub due to the COVID-19 pandemic and therefore found it easy to incorporate into their hygiene routine. In households where handwashing was already common practice, ABHR was a convenient addition:

It is easily accessible … BabyGel is better [than soap]. (M02)

##### Challenges and physical discomfort in using ABHR

Despite these advantages, some participants also described the challenges associated with ABHR use. When hands were visibly dirty the participants had to use water before the ABHR, which took more time. Some participants also misunderstood the instructed procedures, or received insufficient training, using water and/or soap more often than necessary.

She [health worker] told me to first wash my hands with soap, then use sanitizer [ABHR] again and then use soap and water … That is why I preferred soap. (M07)

Although ABHR was generally reported to have a pleasant effect on users’ hands, mothers and household members also experienced negative effects, mainly pain related to ABHR used on wounds. They said this was difficult to avoid, as they often had cuts on their hands. However, even though this was perceived as a burden, it did not deter them from using ABHR:

I had a cut on my hand and after doing my work I sanitized, and it pained me. (M06)

#### Ethicality—fit of ABHR use with participants’ values, norms or beliefs

##### Negotiating ABHR against religious, spiritual and cultural beliefs

Although ABHR use was generally considered acceptable and compatible with participants’ values and beliefs, concerns related to religion, spirituality and cultural norms were also described. Muslim and Christian participants could see ingesting alcohol as bad or forbidden. However, even though ABHR smelled like alcohol, mothers and household members generally considered it acceptable due to its medical purpose and because they had seen it being used in religious settings. Still, some community members remained sceptical and refused to use ABHR because of the alcohol content:

I saw it being used even at the mosques … I then had to change my mindset that it is okay to use sanitizer [ABHR] … Yet, some people in our area are Muslims and even when you asked some people to use sanitizer [ABHR], they refused saying ’I cannot use alcohol to wash my hands’. (M06)

Beyond concerns related to alcohol, some participants also expressed fears that ABHR might affect the baby spiritually. A few participants were afraid that ABHR and other trial products were ‘illuminati’, a term locally used to denote association with the devil or malevolent spiritual forces. They tried to make sense of why they had received free products and questioned how the BabyGel trial would benefit from their participation. Some mothers and household members therefore feared that ‘the devil’ was involved in the BabyGel trial and that they or their baby might be sacrificed. Rumours circulating in the community reinforced these concerns:

We got some fear when people told us that these things you people give us are bad … that they are from the river [underground, where the devil lives] and they will sacrifice us. (M09)

In Uganda, certain cultural practices may conflict with the use of ABHR in newborn care. While most mothers and household members did not report any concerns related to hand hygiene, one mother noted a belief within a specific cultural group that washing hands prior to touching a newborn could be harmful to the child. This belief was reported in a cultural group associated with traditional rites of passage related to reproduction and circumcision.

In the Gishu culture … Telling someone to sanitize before carrying the baby is like taking away blessings from the hands, so someone told me it is bad luck to wash hands before carrying the baby. (M07)

#### Intervention coherence—understanding what ABHR is for and how to use it correctly

##### Hands as a route of infection to a fragile newborn

Overall, mothers and household members reported a good understanding of how and when to use ABHR. They described using it before touching or breastfeeding the baby, after toilet visits, before eating, after household or garden work and after shaking hands. When hands were visibly soiled, they reported washing with water and soap before using ABHR.

You wash the hands with water first to remove the mud, then you apply BabyGel in the hands … You touch the baby when the hands have dried. (HM01, H03)

There was an understanding that dirt and germs could cause infections, particularly in newborns perceived as fragile. Likewise, the pregnant woman was perceived as fragile, together with the baby in the womb. Hands were perceived as a route of transmission between contaminated environments and the baby, and using ABHR was viewed as a way to interrupt this transmission.

Without hand hygiene, it is possible to transmit germs from the hands to the baby’s body, yet their immunity is still weak. (HM01, H05)

In addition to using ABHR on the hands, some participants also used it for unintended purposes, such as adding it to laundry for fragrance, treating skin conditions, killing insects or cleaning dirty surfaces.

I heard that when you have pimples on the face, once you use it on a skin with pimples, all the pimples disappear. (HM01, H07)

#### Opportunity costs*—*time, effort or other activities given up to use ABHR

##### Time costs and everyday inconveniences with ABHR use

Participants described several opportunity costs associated with ABHR use. Waiting for hands to dry before touching the baby was sometimes described as time-consuming, particularly when the infant was crying, leading some to skip hand hygiene in urgent situations:

When I am washing clothes and my baby starts to cry and then I think about having the hands dry, use sanitizer [ABHR] and then wait for them to dry before touching the baby, I felt like that was time wasting … so that is why I would just go and carry the baby without cleaning hands. (M08)

Some also avoided ABHR when cooking or eating fearing that it could contaminate food and cause harm if ingested, or ignite near a flame. They therefore preferred water and soap at these times.

I was told that after sanitizing, I should not eat … I should wash hands again with soap, dry the hands, and then eat. (M06)

##### Social costs and strained relationships with visitors

ABHR use also generated social tensions between the participants and visitors. Visiting family members or neighbours sometimes refused to use the ABHR before touching the newborn, creating conflict. Some mothers also reported that visitors had stopped coming over after being asked to use ABHR before touching the baby.

I have faced resistance from the elderly neighbours who stopped coming to visit the baby because they did not want to hand sanitize … they assured me they feared using hand sanitizer. (M05)

#### Perceived effectiveness—belief that using ABHR prevents infection or improves health

##### Belief that ABHR protects the health of baby and family

Participants generally believed that ABHR prevented disease and protected both themselves and their baby. Many attributed their child’s good health to ABHR use preventing disease, both before and after birth. Some mentioned that after exiting the trial and having stopped using ABHR, their baby fell sick more often. They believed that this benefit extended to the wider household and attributed their family’s good health to ABHR use.

I felt safe knowing that I will not get any disease because I know that all the germs are dead … I realized the baby was healthy and never sickly because she used to use the sanitizer [ABHR] before touching the baby. (HM03, H10)

##### Doubt about the need for and effectiveness of ABHR

Other community members were more sceptical about ABHR and its effect on their health. They questioned the need for ABHR arguing that people had handled babies without hand rub for generations and had survived nonetheless. Some community members also believed that their destiny was already decided by a higher power and using ABHR would not change their destiny:

Even if you use sanitizer [ABHR], you can still die if you are meant to die. (M04)

#### Self-efficacy—confidence in one’s ability to use ABHR properly and consistently

##### Building new hygiene routines that were not always sustained

Many participants described changes in hygiene behaviour following the intervention. Although some had recently used hand rub for COVID-19 infections, the participants emphasised that using ABHR was an improvement in their hygiene routine. After completing the trial period some continued using remaining ABHR before touching their baby:

You get used to using it and it feels like you have to use it all the time … If you have not used sanitizer [ABHR], you feel like something is missing. (M08)

Many participants without remaining ABHR reported using soap and/or water instead before touching the baby. They indicated that hand hygiene prior to newborn contact had not been common practice before the BabyGel trial and that their routines had since changed, reflecting a newly perceived need to handle the infant only with clean hands:

Previously, we never cleaned hands before carrying children … I learned that you have to first wash hands before touching the baby. (HM02, H02)

The poster promoted ABHR use by serving both as a reminder and as a guide when mothers and household members felt uncertain or forgot the correct procedure. Training sessions strengthened their confidence in using ABHR correctly by allowing enough time to absorb the information, opportunities to ask questions and follow-up sessions to reinforce their knowledge. Mothers also trained household members and visitors, and participants reminded each other to use the ABHR before touching the baby.

When I looked at it [the poster], I was able to remember that I have to sanitize before touching the baby … Every time they [neighbours] came to the house to carry the baby and looked at the poster, they also remembered. (M07)

Although confidence in using ABHR was generally high, consistent use was not always maintained, particularly in stressful situations:

The baby was crying, and I just went to carry the baby before using sanitizer [ABHR]. (M04)

## Discussion

This study investigated the acceptability of the ABHR product and found that the BabyGel intervention was generally well accepted among mothers, caregivers and household members during the first 3 months after birth. ABHR was described as feasible and easy to use, although some participants reported inconvenience and fears of bodily harm or spiritual consequences. We discuss factors enhancing and limiting acceptability across the TFA constructs, situating our findings within existing literature on ABHR in community settings.

### Factors enhancing acceptability

Participants largely expressed positive attitudes towards ABHR and believed it effectively prevented disease by eliminating pathogens and protecting themselves and their children. Perceived reductions in illness reinforced these beliefs. ABHR was associated with bodily comfort, cleanliness. Its limited availability in these communities also contributed to perceptions of elevated social status and exclusivity. It was generally considered low-effort, accessible and time-saving compared to handwashing with soap and water. Similar findings from rural Bangladesh highlight convenience and usability as key drivers of acceptability [11].

The user-friendliness of ABHR, combined with training sessions and the poster strengthened self-efficacy. The poster served as a practical reminder and reference tool, consistent with findings from the BabyGel pilot study [18], enhancing participants’ confidence, especially when they were uncertain about ABHR practices.

Recruitment during the COVID-19 pandemic probably influenced acceptability. Heightened fear of infection and increased public awareness of infection prevention may have amplified perceptions of ABHR’s protective value and willingness to adopt hand hygiene practices, which may have limited transferability to non-pandemic contexts. Widespread hygiene-awareness campaigns during the pandemic, for example, may have contributed to the level of intervention coherence and self-efficacy we observed [25]. Religious authorities in Uganda also promoted hand rub use [26], probably contributing to its ethical acceptability. Despite initial concerns about alcohol content, both Muslim and Christian participants generally accepted ABHR for medical purposes. Although some participants used ABHR for unintended purposes (e.g. laundry), they generally understood its primary function. Using household items for purposes other than those intended is widely documented in community-based disease prevention, for example the use of mosquito nets for fishing [27].

Importantly, several participants reported continued hand hygiene practices after the intervention had ended, suggesting that temporary exposure to ABHR and accompanying hygiene education may have contributed to sustained behavioural change beyond the trial period.

### Factors limiting acceptability

Participants raised concerns about ABHR use, particularly regarding potential harm to themselves or their child. Precautionary practices, such as rinsing hands after use before cooking or breastfeeding, were adopted to mitigate perceived risks. Scepticism from community members also emerged, including beliefs that health outcomes were predetermined by faith and not influenced by preventive measures.

Culture shapes social norms, beliefs and behaviours [28]. While Ugandan society is largely shaped by Christianity and Islam, traditional belief systems remain influential. Studies from Uganda and Kenya show that stillbirth is sometimes interpreted through traditional beliefs, such as witchcraft [29,30]. Similarly, some participants expressed spiritual concerns about ABHR use or suspicions regarding its distribution, including conspiracy narratives linking free ABHR to occult or covert organisations. Community resistance to medical research has previously been documented [31], including in a Kenyan HIV trial where some participants associated incentives with occult practices [32]. The rumours may reflect broader community uncertainty regarding externally introduced interventions and free products. Although primarily expressed by other community members, they nevertheless shaped participants’ experiences, illustrating how community perceptions may shape the social context in which interventions are introduced and used.

Physical discomfort, including nausea and pain, when ABHR was applied to cuts reduced acceptability. Some participants applied ABHR to wounds despite instructions not to do so. Others washed hands unnecessarily before or after ABHR use, suggesting partial misunderstanding of recommended ABHR practices. These findings highlight the importance of clear and contextually appropriate hygiene education alongside product provision.

Beyond physical discomfort, the use of ABHR was associated with social and relational costs. Several participants described situations in which visitors refused to use ABHR before touching the newborn, placing caregivers in difficult situations where enforcing hygiene practices risked offending visitors and damaging social relationships. The desire to avoid conflict may undermine fidelity to the intervention.

An important finding was that the perceived benefits of ABHR sometimes conflicted with existing social norms and relationships. Asking visitors to sanitise their hands could be interpreted as questioning their cleanliness, while the prestige associated with ABHR could invite accusations of showing off, or create social distance. These findings suggest that perceived effectiveness alone may not be sufficient to ensure uptake and sustained use if an intervention conflicts with existing social relationships and community norms.

Participants sometimes found that waiting for their hands to dry before caring for the baby interfered with care-giving. Participants’ accounts of the time ABHR required were ambiguous: although ABHR was viewed as faster than soap and water, waiting for hands to dry or combining ABHR with handwashing when hands were visibly dirty was sometimes considered time-consuming. Similar preferences for soap and water when hands are visibly soiled have been reported in Bangladesh [11] and among midwives in Sierra Leone [13].

Underlying economic constraints were also visible: households had limited access even to soap. Commercially available ABHR is generally more expensive than soap and water and has more limited household applications. The affordability and sustainability of ABHR provision outside a trial setting therefore require further research, including assessment of implementation approaches, such as targeted subsidies or provision around childbirth. Sustained uptake may depend on subsidised or otherwise targeted delivery strategies, particularly in the poorest communities, where household poverty is itself a risk factor for early-infant infection [33].

Overall, our findings align with previous ABHR acceptability studies [10,11,13,34]. Some of the above-mentioned obstacles to using ABHR in the neonatal and infant period might have contributed to the negative trial results. Furthermore, user compliance and fidelity to correct ABHR use remain unknown and may have been inadequate.

### Strengths and limitations

The qualitative assessment of an intervention’s acceptability within a trial provided insight into participants’ experiences, trial-specific challenges and the relationship between trial context and acceptability. Sekhon’s acceptability framework provided a structured, theory-informed approach, strengthening transparency and interpretability. Interviews were deliberately conducted in participants’ homes by trained research assistants using participants’ preferred languages, which probably enhanced trust and openness. Semi-structured interview guides enabled flexible discussions while ensuring coverage of key topics.

This study had limitations. Direct participant observations during the trial period were not feasible because participants were recruited only after they had completed follow-up to avoid influencing trial outcomes. Observations from households and training sessions could have clarified how ABHR was used and whether reported behaviours aligned with daily routines, including consistency of use, interpretation of instructions and visitor interactions with newborn hygiene practices.

Interviews were conducted only with households that had completed the intervention. Community members from other households were excluded to minimise the risk of influencing participants who had not yet received the intervention or were still enrolled. Consequently, we did not capture perspectives from participants who were actively engaged with the intervention, which may have provided insight into early challenges, evolving perceptions and integration of hand hygiene practices during the trial period.

Mirroring the trial population, participants were predominantly women. Purposive sampling ensured socio-demographic variation and representation across the catchment area. We aimed to include men among household members, but data collection was restricted to daytime due to logistical constraints. As a result, women were more often encountered at home, whereas male caregivers, who were more often absent during these hours, were underrepresented, potentially introducing non-response bias [35]. Participants were identified with assistance from village health teams, which may have introduced selection bias, as individuals better known to the village health teams, or those with more favourable experiences, may have been more readily identified or more inclined to participate.

Social desirability bias relating to the trial product may also have shaped participants’ accounts [36]. Participants may have expected social and monetary benefits from participation, which may have encouraged more favourable accounts of the intervention. This may have contributed to over-reporting of positive experiences and of adherence to recommended hygiene practices.

Lastly, working across languages introduced risk of information being lost or misinterpreted during interviews and analysis. Interviews were conducted in participants’ native languages and then translated into English. Translation prior to analysis can introduce bias and reduce nuance [37], particularly when interpreting culturally embedded concepts, idioms and expressions. At times, assistants did not speak a participant’s preferred language, which may have created power imbalances and further increased the risk of lost nuances or misinterpretation.

## Conclusion

The BabyGel intervention was generally well accepted among mothers and household members, who valued ABHR’s convenience, protective effect and social benefits. However, concerns related to safety, cultural beliefs, social dynamics and occasional inconvenience also influenced its use. Successful adoption of ABHR may require culturally adapted education, community engagement and reinforcement of correct use, particularly in settings where local beliefs and social norms influence perceptions of hygiene interventions. Importantly, several participants reported sustained hand hygiene practices beyond the intervention period, raising the question of whether short-term, intensive exposure around birth may contribute to lasting behavioural change. Future research should examine what sustains ABHR adherence beyond product acceptability, including structural constraints, household decision-making and community-level norms.

## Supplementary Material

Main tables_v2.docx

Supplementary file 1_BabyGel Poster_before after birth.docx

Supplementary file 3_COREQ_Checklist_Global Health Action 3.docx

Supplementary file 2_Interview guides.docx

## Data Availability

The data that support the findings of this study are available from the corresponding author [HHT] upon reasonable request.
