# Supplementary material for: Acceptability of alcohol-based hand rub during neonatal care in rural Ugandan households: a qualitative study nested within the BabyGel trial
Source: Glob Health Action. 2026 Sep 4;19(1):2727791. doi: 10.1080/16549716.2026.2727791 (PMC13552334; doi:10.1080/16549716.2026.2727791)
Supplement: Supplementary file 1 [file ZGHA_A_2727791_SM0417.docx]

**Interview guide – Interview with postpartum women**

# ***Specific objective:*** *Explore how the ABHR intervention was perceived by adult household members; what aspects of the intervention were seen as barriers and facilitating during the 3 months follow-up.*

**Introduction**

Hello, my name is ……………., and I am a researcher from the………….. . I am part of a project titled: “Am I too dirty to touch my baby? Providers’ and users’ perceptions of a hand hygiene intervention in the neonate period: A qualitative study in Eastern Uganda” whose PI is Heidi Thorsdalen, from the University of Bergen in Norway. We would like to understand your experience with BabyGel sanitiser, poster that you were given and the training on how to use the sanitiser. We would like to listen to what you have to say about this and to use your experience to improve other participants experiences and future interventions. The interview will take about 40-50 minutes. We would like to ensure we capture everything you say, so we will use a recorder as we informed in the consent forms, and all recordings will be deleted after the research is completed. We will not disclose or use your personal details like name in our report, so what you say in this interview will not be possible to trace back to you. Participation is voluntary and you can withdraw at any time. Would you like for us to go on or do you have any questions before we start?

**Information about the postpartum woman**

1. Can you tell me about yourself (*age, what you do during daytime (occupation), number of household members, age of each household member?*) (*Fill in form and ask specific questions*)

**Perceptions on the training**

2. What has been your experience with getting visited by the trainers?

- How did you feel when they came home to you? A bit scared?
- How has the experience been for the rest of the household members? (*The children, husband etc.)*
- How did the rest of the household experience the training before and after birth? How was it for them to be told not to use before birth and to use after birth? (*Look into especially the children’s perception on don’t using and then using after. What did they tell the children?*)
- Any disagreements between the household members?
- Did the other household members listen to you when you told them to use ABHR?

3. What did you learn from the training?

- Was the information new to you or do did you know it from before?
- Had you used hand sanitizer before entering BabyGel study?

4. Were there any difficulties related to the training?

- If so, what were they?

5. Anything you liked about the training?

- If so, what?

6. How was your experience with the phone calls?

- How were the phone calls compared to the face-to-face visits?
- What did you prefer? Why?

7. What are your thoughts on the amount of training you received? (*Discover if she would have wished to have more or less visits)*

- Do you feel you got enough time with the trainer?
- How many times were you trained?
- Did you sometimes miss the training or that the trainer did not show up? Delays?

8. Is there anything that can be improved with the training?

- Could you please share your thoughts on improvements that could be made?
- What changes would you have liked to do with the training?
- Any complaints?

**Perceptions on the poster**

9. How was your experience with the poster? Please explain

- Is the anything difficult to understand about the poster?
- What do you think about the pictures on the poster? (*Was it clear?)*
- What do you think about the text on the poster? (*Was it clear?)*
- Do you have any suggestions to make it more understandable? (*How could it be more understandable?*). Improvements?
- How was it to store the poster? Where did you hang it? Did you have any challenges?
- Any complaints?

**Perceptions on the BabyGel (ABHR)**

10. How has your experience been with using the BabyGel (ABHR)?

- How did you feel using it? (*Do they feel important*?)
- When did you use the BabyGel (ABHR)? To which occasions?
- Tell about a time when it was problematic to use BabyGel (ABHR). (*Times where she preferred water and soap over ABHR?*)
- What were your thoughts on hand sanitizer before entering and after? Was there any fear?
- What makes it difficult to use BabyGel (ABHR)?
- Any complaints about the BabyGel?

11. Tell about what you liked with the BabyGel (ABHR)? Tell about what you disliked with the BabyGel (ABHR)

- Was it easy to use?
- Which BabyGel bottle did you prefer from the three different you got?
- How did you find the smell of the BabyGel? Has your religion affected your use of BabyGel (ABHR)?

12. What can be improved with the BabyGel (ABHR)?
13. Did you get visitors to use the BabyGel (ABHR)?

- What were their responses?
- If they did not use, why/why not?

14. Would you like to continue to use the BabyGel (ABHR) and if so, why?

- Do you think you would use it for potential later pregnancies?

15. Have there been any incidents, such as accidents or burns?

- Where you told about any precautions?

**Covid-19 impact on the experience**

16. How did covid-19 affect your BabyGel-trial experience

- Probe on: hygiene practices before and during the COVID-19 pandemic
- Probe on: ABHR experiences before and during the COVID-19 pandemic
- How did it affect your use of BabyGel (ABHR)?
- What was your experience with phone calls instead of face-to-face visits? How was it for you? [For those who experienced the lockdown]
- Have it affected the quality of the training you received?

17. Is there anything else you would like to share?

**Interview guide – Group interview with household**

# ***Specific objective:*** *Explore how the ABHR intervention was perceived by adult household members; what aspects of the intervention were seen as barriers and facilitating during the 3 months follow-up.*

Hello, my name is ……………., and I am a researcher from the………….. . I am part of a project titled: “Am I too dirty to touch my baby? Providers’ and users’ perceptions of a hand hygiene intervention in the neonate period: A qualitative study in Eastern Uganda” whose PI is Heidi Thorsdalen, from the University of Bergen in Norway. We would like to understand your experience with BabyGel sanitiser, poster that you were given and the training on how to use the sanitiser. We would like to listen to what you have to say about this and to use your experience to improve other participants experiences and future interventions. The interview will take about 40-60 minutes. We would like to ensure we capture everything you say, so we will use a recorder as we informed in the consent forms, and all recordings will be deleted after the research is completed. We will not disclose or use your personal details like name in our report, so what you say in this interview will not be possible to trace back to you. Participation is voluntary and you can withdraw at any time. Would you like for us to go on or do you have any questions before we start?

**Information about the household**

18. (*Fill in form and ask specific questions*)

**Perceptions on the training**

19. What has been your experience with getting visited by the trainers?

- How did you feel when they came home to you? A bit scared?
- How has the experience been for the rest of the household members? (*The children, husband etc.)*
- How did you/the rest of the household experience the training before and after birth? How was it for you (except the mother) to be told not to use before birth and to use after birth? (*Look into especially the children’s perception on don’t using and then using after. What did they tell the children?*)
- Any disagreements between you/the household members?

20. What did you learn from the training?

- Was the information new to you or do did you know it from before?
- Had you used hand sanitizer before entering BabyGel study?

21. Were there any difficulties related to the training?

- If so, what were they?

22. Anything you liked about the training?

- If so, what?

23. How was your experience with the phone calls?

- How were the phone calls compared to the face-to-face visits?
- What did you prefer? Why?

24. What are your thoughts on the amount of training you received? (*Discover if they would have wished to have more or less visits)*

- Do you feel you got enough time with the trainer?
- How many times were you trained?
- Did you sometimes miss the training or that the trainer did not show up? Delays?

25. Is there anything that can be improved with the training?

- Could you please share your thoughts on improvements that could be made?
- What changes would you have liked to do with the training?
- Any complaints?

**Perceptions on the poster**

26. How was your experience with the poster? Please explain

- Is the anything difficult to understand about the poster?
- What do you think about the pictures on the poster? (*Was it clear?)*
- What do you think about the text on the poster? (*Was it clear?)*
- Do you have any suggestions to make it more understandable? (*How could it be more understandable?*). Improvements?
- How was it to store the poster? Where did you hang it? Did you have any challenges?
- Any complaints?

**Perceptions on the BabyGel (ABHR)**

27. How has your experience been with using the BabyGel (ABHR)?

- How did you feel using it? (*Do they feel important*?)
- When did you use the BabyGel (ABHR)? To which occasions?
- Tell about a time when it was problematic to use BabyGel (ABHR). (*Times where they preferred water and soap over ABHR?*)
- What were your thoughts on hand sanitizer before entering and after? Was there any fear?
- What makes it difficult to use BabyGel (ABHR)?
- Any complaints about the BabyGel?

28. Tell about what you liked with the BabyGel (ABHR)? Tell about what you disliked with the BabyGel (ABHR)

- Was it easy to use?
- Which BabyGel bottle did you prefer from the three different you got?
- How did you find the smell of the BabyGel? Has your religion affected your use of BabyGel (ABHR)?

29. What can be improved with the BabyGel (ABHR)?
30. Did you get visitors to use the BabyGel (ABHR)?

- What were their responses?
- If they did not use, why/why not?

31. Would you like to continue to use the BabyGel (ABHR) and if so, why?

- Do you think it could be used for potential later pregnancies?

32. Have there been any incidents, such as accidents or burns?

- Where you told about any precautions?

**Covid-19 impact on the experience**

33. How did covid-19 affect your BabyGel-trial experience

- Probe on: hygiene practices before and during the COVID-19 pandemic
- Probe on: ABHR experiences before and during the COVID-19 pandemic
- How did it affect your use of BabyGel (ABHR)?
- What was your experience with phone calls instead of face-to-face visits? How was it for you? [For those who experienced the lockdown]
- Has it affected the quality of the training you received?

34. Is there anything else you would like to share?

**Date:**

**Associated hub: Duration of interview:**

**Number of adult household members: Number of children:**

| **Household member** | **Age** | **Gender** | **Occupation** | **Level of education** | **Religion** | **Marital status** | **Phone number** |
| --- | --- | --- | --- | --- | --- | --- | --- |
|  |  |  |  |  |  |  |  |
|  |  |  |  |  |  |  |  |
|  |  |  |  |  |  |  |  |
|  |  |  |  |  |  |  |  |
|  |  |  |  |  |  |  |  |
|  |  |  |  |  |  |  |  |
|  |  |  |  |  |  |  |  |
