# Supplementary material for: Acceptability of alcohol-based hand rub during neonatal care in rural Ugandan households: a qualitative study nested within the BabyGel trial
Source: Glob Health Action. 2026 Sep 4;19(1):2727791. doi: 10.1080/16549716.2026.2727791 (PMC13552334; doi:10.1080/16549716.2026.2727791)
Supplement: Supplementary file 3 [file ZGHA_A_2727791_SM0419.docx]

**BabyGel Poster**

Supplementary Figure 1A: BabyGel Poster before birth

Supplementary Figure 1B: BabyGel Poster after birth
