# Supplementary material for: Acceptability of alcohol-based hand rub during neonatal care in rural Ugandan households: a qualitative study nested within the BabyGel trial
Source: Glob Health Action. 2026 Sep 4;19(1):2727791. doi: 10.1080/16549716.2026.2727791 (PMC13552334; doi:10.1080/16549716.2026.2727791)
Supplement: Supplementary file 4 [file ZGHA_A_2727791_SM0420.docx]

**Main tables**

Table 1: Sociodemographic characteristics of study participants (N=34).

| **Characteristics** | **Mothers (*n*=20)** | **(%)** | **Household members (*n*=14)** | **(%)** | **Total (*N*=34)** | **(%)** |
| --- | --- | --- | --- | --- | --- | --- |
| **Age (Years)** |  |  |  |  |  |  |
| 17-24 | 8 | (40) | 4 | (29) | 12 | (35) |
| 25-39 | 12 | (60) | 5 | (36) | 17 | (50) |
| 40-64 | 0 | (0) | 4 | (29) | 4 | (12) |
| Missing | 0 | (0) | 1 | (7) | 1 | (3) |
| **Education (Years)** |  |  |  |  |  |  |
| 0-7 | 14 | (70) | 8 | (57) | 22 | (65) |
| 8-13 | 4 | (20) | 5 | (36) | 9 | (27) |
| Above 13 | 2 | (10) | 0 | (0) | 2 | (6) |
| Missing | 0 | (0) | 1 | (7) | 1 | (3) |
| **Religion** |  |  |  |  |  |  |
| Muslim | 11 | (55) | 7 | (50) | 18 | (53) |
| Christian | 9 | (45) | 7 | (50) | 16 | (47) |

Table 2: Themes reflecting mothers’ and household members’ perceptions of the BabyGel intervention by TFA categories.

| ***Construct*** | ***Definition*** | ***Theme*** |
| --- | --- | --- |
| **Affective attitude** | How an individual feels about an intervention | Valuing ABHR for comfort, cleanliness and status  Fear and mistrust of ABHR as potentially harmful |
| **Burden** | The perceived amount of effort that is required to participate in an intervention | ABHR as a convenient alternative to handwashing  Challenges and physical discomfort in using ABHR |
| **Ethicality** | The extent to which the intervention has good fit with an individual’s value system | Negotiating ABHR against religious, spiritual and cultural beliefs |
| **Intervention coherence** | The extent to which the participant understands the intervention and how it works | Hands as a route of infection to a fragile newborn |
| **Opportunity costs** | The extent to which benefits, profits or values must be given up to engage in the intervention | Time costs and everyday inconveniences with ABHR use  Social costs and strained relationships with visitors |
| **Perceived effectiveness** | The extent to which the intervention is perceived as likely to achieve its purpose | Belief that ABHR protects the health of baby and family  Doubt about the need for and effectiveness of ABHR |
| **Self-efficacy** | The participant’s confidence that they can perform the behaviour(s) required to participate in the intervention | Building new hygiene routines that were not always sustained |
